# Machine Learning Approaches for Integrating Clinical and Radiographic Data in the Early Detection of Osteonecrosis of the Jaw: Machine Learning Approaches for Integrating Clinical Data in Osteonecrosis of the Jaw

**DOI:** 10.31661/gmj.v13iSP1.3623

**Published:** 2024-12-08

**Authors:** Reza Mahmoudi Anzabi, Ashkan Badkoobeh, Majid Nabian, Naghmeh Shenasa, Zahra Khayami, Meysam Mohammadikhah, Fatemeh Abedi Diznab

**Affiliations:** ^1^ Department of Orthodontics, Faculty of Dentistry, Tabriz University of Medical Sciences, Tabriz, Iran; ^2^ Department of Oral and Maxillofacial Surgery, School of Dentistry, Qom University of Medical Sciences, Qom, Iran; ^3^ Instructor of Operating Room Borojen School of Nursing Shahrekord University of Medical Sciences, Iran; ^4^ Department of Endodontics, Private Practice, Shahrekord university of Medical Science, Shahrekord, Iran; ^5^ Department of Restorative, School of Dentistry, Dental Research Center, Hamadan University of Medical Sciences, Iran; ^6^ Department of Oral and Maxillofacial Surgery, School of Dentistry, Alborz University of Medical Sciences, Karaj, Iran; ^7^ Department of Orthodontics, School of Dentistry, Tehran University of Medical Sciences, Tehran, Iran

**Keywords:** Machine Learning, Radiographic Data, Osteonecrosis of the Jaw

## Abstract

Osteonecrosis of the jaw (ONJ) is a severe bone condition characterized by the progressive destruction of the jawbone, often associated with the long-term use of antiresorptive medications such as bisphosphonates. Early detection of ONJ remains a significant clinical challenge due to the subtle onset of symptoms and limitations in current diagnostic methods, which rely on clinical assessment and radiographic imaging. Conventional techniques, such as panoramic X-rays, computed tomography (CT), and magnetic resonance imaging (MRI), often fail to detect early-stage ONJ, delaying diagnosis until more advanced stages. Machine learning (ML) has emerged as a powerful tool for improving the early detection and diagnosis of ONJ by integrating clinical and radiographic data. ML algorithms, including supervised learning methods like random forests, support vector machines, and deep learning models such as convolutional neural networks, are particularly suited for analyzing complex datasets and identifying patterns that are undetectable by traditional methods. These models can enhance the sensitivity and specificity of ONJ detection, potentially leading to earlier interventions and improved patient outcomes.This paper reviews the current state of ML applications in ONJ detection, emphasizing the integration of clinical and radiographic data. It discusses various ML approaches, their potential to improve diagnostic accuracy, and the challenges involved in data integration. Also, this review highlights future directions of ML as a diagnostic tool that has the potential to revolutionize ONJ detection, offering a path toward earlier, more accurate diagnosis and better patient care.

## Introduction

Osteonecrosis of the jaw (ONJ) is a serious bone disorder characterized by the progressive destruction of jawbone tissue due to disrupted blood flow [1]. This condition often arises as a complication in patients receiving long-term bisphosphonate therapy or other antiresorptive drugs, typically prescribed for osteoporosis, metastatic cancers, or bone-related conditions [2]. ONJ typically manifests with symptoms such as exposed necrotic bone, jaw pain, and infections in the oral cavity. If left untreated, it can lead to severe complications, such as pathologic fractures, and greatly impairing quality of life [3][4].

The early detection of ONJ remains a significant clinical challenge due to the insidious onset of the disease [5]. Risk factors for ONJ include bisphosphonate use, prior dental surgery, pre-existing periodontal disease, and systemic conditions like diabetes or immunosuppression [6]. Despite the clear association between these risk factors and ONJ, the disease often goes unnoticed in its initial stages because early symptoms are either non-specific or absent, delaying diagnosis until more advanced bone damage occurs [7]. This diagnostic gap highlights the urgent need for more sensitive detection strategies, particularly during the early stages of disease progression [8].

The current diagnosis of ONJ is typically based on a combination of clinical and radiographic findings. Clinically, healthcare providers look for exposed necrotic bone in the oral cavity, but this symptom usually becomes apparent only in advanced stages [1][8].

Early-stage ONJ, however, often lacks visible clinical markers, further complicating timely diagnosis [5]. Radiographic techniques such as panoramic X-rays, computed tomography (CT), and magnetic resonance imaging (MRI) are the primary imaging modalities used to assess bone health and detect signs of necrosis [9]. CT scans offer more detailed cross-sectional imaging, allowing for the visualization of both cortical and trabecular bone, but they are typically reserved for more advanced cases due to their higher cost and radiation exposure [10].

on the other hand, MRI provides excellent soft tissue contrast and can detect early bone marrow changes, but its availability and expense limit its use in routine diagnostics [11].

Additionally, these imaging modalities are subject to interpretation variability, where the accuracy of diagnosis depends heavily on the expertise of the radiologist or clinician [11][12]. Overall, conventional diagnostic methods are limited in their ability to detect ONJ early, often leading to delays in treatment [5]. Machine learning (ML) has emerged as a transformative tool in healthcare, with the potential to significantly enhance the early detection and diagnosis of osteonecrosis [13]. ML algorithms are designed to learn from large datasets, identifying patterns and relationships that may be undetectable by traditional methods [14].

By processing and analyzing both structured clinical data and unstructured radiographic images, ML models can identify subtle features of ONJ in its early stages [11]. Moreover, ML algorithms can continuously improve over time, as they are exposed to more data, further enhancing their predictive power [13].

The objective of this review is to critically assess the role of ML in integrating clinical and radiographic data for the early detection of ONJ, limitations, and potential applications. Also, it will explore the challenges associated with the integration of multimodal data and offer insights into future directions for research in this evolving field.

## 1. Clinical and Radiographic Data

In the diagnosis of ONJ, both clinical and radiographic data play essential roles, each offering unique insights that are critical for early detection or prediction [1][5]. However, histopathology images or genetic polymorphism data have been used in some recent studies [15][16].

## 2. Clinical Data

Clinical data for ONJ encompass a wide range of patient-specific factors [1]. Major elements include patient medical records, particularly regarding the use of antiresorptive medications such as bisphosphonates or denosumab, which are strongly linked to ONJ development [6][8] Additional risk factors such as recent dental procedures (e.g., extractions or implants), trauma to the jaw, immunosuppressive conditions, and concurrent treatments like chemotherapy or corticosteroids also contribute to the clinical risk profile [3][8]. Symptoms in ONJ often evolve gradually, with early signs including mild jaw pain or discomfort, swelling, and loose teeth. More advanced stages are characterized by exposed necrotic bone, non-healing wounds, and localized infections [1][8] However, early-stage ONJ often lacks overt clinical symptoms, complicating the diagnostic process and highlighting the importance of combining clinical assessments with imaging data for a more comprehensive evaluation [5].

## 3. Radiographic Data

Radiographic imaging serves as a critical tool for assessing the structural changes in the mandible associated with ONJ. Common imaging modalities include panoramic X-rays, computed tomography (CT), and magnetic resonance imaging (MRI) [11]. Each technique provides different diagnostic advantages, yet all are valuable for detecting bone alterations. Panoramic X-rays are frequently used as an initial imaging tool due to their wide availability and ability to visualize the entire mandible in one scan [17]. However, this modality has limitations in sensitivity, particularly for detecting early bone changes [11].

CT scans offer more detailed cross-sectional images of the bone, allowing for better visualization of both cortical and trabecular bone structures. This technique is particularly useful for identifying subtle features like early osteosclerosis or osteolysis that may not be evident on plain radiographs [10]. MRI, while less commonly employed due to its high cost and limited availability, excels in detecting early changes in the bone marrow that precede visible bone necrosis, offering a higher sensitivity for detecting early-stage ONJ [11]. Major diagnostic radiographic features of ONJ include areas of radiolucency, radiopacity, and the presence of sequestra [18].

## 4. Data Integration Challenges

The integration of clinical and radiographic data for ONJ diagnosis presents several technical challenges, primarily because these two types of data are fundamentally different in both format and scale [19]. Clinical data, often structured as patient history, risk factors, and symptom descriptions, is typically categorical or numerical, whereas radiographic data is unstructured, consisting of high-dimensional image files that require preprocessing for analysis [20]. This disparity complicates the direct fusion of these data types into ML models designed to enhance early ONJ detection [13]. Furthermore, radiographic images need to undergo extensive preprocessing, including segmentation and feature extraction, to isolate the relevant areas of interest, such as bone abnormalities [14].

On the other hand, clinical data is often incomplete or inconsistent, which can lead to gaps in the information provided to the model [19]. Another major challenge is aligning the timing of the data. Clinical symptoms may appear at different stages of the disease compared to radiographic changes, making it difficult to synchronize the data inputs accurately. Moreover, the integration process must account for the fact that clinical and radiographic features may not correlate directly with disease severity or progression [19][21].

ML models that aim to combine these two data streams must be capable of handling these discrepancies and extracting meaningful patterns from diverse and often discordant datasets [14]. Developing robust algorithms that can simultaneously analyze clinical risk factors and radiographic features requires sophisticated data fusion techniques capable of managing these complexities [20]. Table-1 highlights primary diagnostic features and evaluates their potential for integration into ML models to improve detection and diagnosis.

Thus, while the combination of clinical and radiographic data offers the potential for more accurate and earlier ONJ diagnosis, the technical and methodological challenges associated with merging such heterogeneous data must be addressed to fully realize the benefits of an integrated approach.

## 5. Overview of ML Approaches

**Table:** Table1. Clinical and Radiographic Features of ONJ and their Suitability ML Applications

**Feature**	**Description**	**Role in Diagnosis**	**ML Suitability**
**Clinical**
Mandibular Pain	Pain in the jaw, often exacerbated by pressure or chewing.	Primary symptom prompting clinical investigation of ONJ.	Moderate - Clinical symptoms are subjective but can be incorporated into multi-modal ML models.
Bone Exposures (Clinical)	Visible bone exposed in the oral cavity without healing over a long period.	A clear indication of ONJ in the clinical setting is often used as a primary diagnostic marker.	High - Easily identifiable in clinical records and photographs for ML classification.
**Radiographic**
Osteolysis	Destruction of bone structure is typically seen in the advanced stages of ONJ.	Helps confirm the extent of bone damage and progression of ONJ.	High - Clear radiographic marker, frequently used in ML models for bone destruction detection.
Osteosclerosis	Abnormal hardening of the bone may indicate a reactive process to necrosis.	Indicator of chronic disease or healing responses, aiding in staging ONJ severity.	High - Detectable in radiographs, useful in distinguishing stages of ONJ.
Cortical Erosion	Thinning or wearing away of the cortical bone layer in the mandible.	Early signs of bone weakening and necrosis in ONJ progression.	Moderate - Requires high-quality imaging, but is useful for early detection algorithms.
Focal Sclerosis	Increased bone density is seen in localized areas, often near necrotic regions.	A sign of localized bone death, used to differentiate between stages of necrosis.	High - Often used in radiographic analysis for ONJ detection in ML models.
Sequestrum	Dead bone fragments detached from living bone, visible in radiographs.	Indicates severe necrosis and poor prognosis.	High - Clear radiographic marker for severe ONJ, useful in advanced detection models.
Persistent Alveolar Socket	Non-healing extraction socket after tooth removal, which can indicate impaired healing.	Suggests compromised bone healing, a critical indicator of early ONJ.	Moderate - Radiographic evidence can be subtle, but useful for early-stage ONJ prediction models.
Inferior Alveolar Canal Enhancement	Radiographic enhancement along the canal, suggests nerve involvement or advanced disease.	Helps distinguish between ONJ and other mandibular pathologies.	Moderate - Requires precise imaging, but is useful for advanced ONJ detection.

ML has shown great potential in advancing the early detection and diagnosis of ONJ by enabling the analysis of large datasets and identifying patterns that may be difficult for clinicians to detect using traditional methods [13]. Several ML approaches, including supervised, unsupervised, deep learning, and multimodal techniques, have been explored for ONJ, each offering unique advantages in processing clinical and radiographic data.

### 5. 1. Supervised Learning

Supervised learning is a commonly used ML approach where the model is trained on labeled data, with both input features and their corresponding outcomes provided. Supervised learning models like random forests, support vector machines (SVMs), and neural networks (NNs) have been employed to predict ONJ occurrence based on clinical and radiographic data [14][22].

Random forests are ensemble-based models that combine multiple decision trees to improve predictive accuracy and handle large, heterogeneous datasets [23]. They have been used to assess structured clinical data, such as patient risk factors and medical history, to predict the likelihood of ONJ development [24].

SVMs are another popular supervised learning technique that separates data into different classes using hyperplanes [25]. For ONJ detection, SVMs can classify patients into "high-risk" or "low-risk" groups based on clinical and radiographic input. They are especially effective when dealing with small datasets, which is often the case with rare conditions, and are robust against overfitting in high-dimensional spaces [14][24].

NNs, especially shallow networks, are capable of capturing complex, non-linear relationships between clinical features and ONJ risk [26][27]. These models can be trained to predict disease presence based on clinical data inputs but have been less commonly applied to structured data in ONJ compared to their deep-learning counterparts [28].

### 5. 2. Unsupervised Learning

Unsupervised learning techniques, which work with unlabeled data, are particularly useful in scenarios where clear outcomes are not predefined, making them valuable for exploratory analysis [29].

Clustering algorithms such as k-means or hierarchical clustering can be applied to group patients based on similarities in their clinical and radiographic profiles [30]. These techniques allow for the identification of subgroups of patients who may be at higher or lower risk for ONJ, based on common clinical features or imaging characteristics. Clustering can also help uncover hidden patterns in patient data [24][30].

### 5. 3. Deep Learning

Deep learning, a subset of machine learning that uses NNs with multiple layers, has revolutionized the analysis of complex data types, particularly in medical imaging [31]. For osteonecrosis detection, convolutional NNs (CNNs) have been instrumental in analyzing radiographic images [27].

NNs are designed to automatically learn hierarchical features from images, making them highly effective at detecting subtle bone changes that may indicate early stages of ONJ [13][27]. These models can process radiographic images like panoramic X-rays, CT, or MRI scans to identify key diagnostic features [27][32]. Advanced forms of CNNs, such as U-Net models, have also been used for segmentation tasks, where the goal is to delineate areas of necrotic bone from healthy tissue [15]. This automated segmentation helps clinicians focus on regions of interest in complex radiographic images, providing a more detailed assessment of ONJ progression [15][33].

### 5. 4. Multimodal Machine Learning

Multimodal machine learning integrates multiple data types primarily clinical and radiographic data into a unified predictive model [20]. Multimodal ML approaches are designed to address the limitations of single-modality models by combining structured clinical data with unstructured radiographic images, allowing for a more comprehensive analysis [20][34].

• Hybrid models: These models incorporate both clinical and radiographic features as inputs, enabling them to leverage the complementary strengths of each data type [35]. In hybrid models, the two data types are processed simultaneously, allowing the model to make more informed predictions. Feature extraction techniques are used to reduce the dimensionality of radiographic images, making them more manageable for analysis alongside clinical data to predict osteonecrosis [36].

• Ensemble models: Ensemble methods, which combine the predictions of multiple different models to improve overall accuracy [37][38]. This method helps to mitigate the weaknesses of any single model by pooling the strengths of multiple approaches, resulting in more robust and reliable predictions [13][39].

## 6. Clinical Applications of ML in ONJ

**Table:** Table2. Comparison of ML Models for the Detection or Prediction of ONJ

**Study**	**Input Data**	**Algorithm**	**Performance Metrics**	**Applied for**
Ariji et al. [40]	Radiographic	NN	Sensitivity:0.88	detection
Gürses et al. [14]	Radiographic	SVM	Accuracy:99.9% Sensitivity:99.8%	detection
Matthies et al. [15]	histopathology image	NN	Accuracy:100% Sensitivity:100%	detection
		RF	AUC: 0.97 Sensitivity: 100%	prediction
Kim et al.[24]	Clinical	ANN	AUC: 0.91 Sensitivity: 100%	
		SVM	AUC: 0.88 Sensitivity:81.8%	
		LR	AUC:0.84 Sensitivity: 100%	
Humbert-Vidan et al., [41]	Clinical and Radiographic	RF	Accuracy: 77%	prediction
Kwack et al. [13]	Clinical and Radiographic	Deep Learning (generalized linear model)	Accuracy: 82% AUC: 0.83	prediction
Choi et al. [16]	estrogen receptor 1 polymorphisms	RF	AUC:0.8	prediction
		SVM	AUC:0.76	
		LR	Accuracy: 75% Sensitivity: 90%	prediction
		SVM	Accuracy: 76% Sensitivity: 96%	
Reber et al. [39]	Clinical and Radiographic	RF	Accuracy: 71% Sensitivity: 77%	
		AdaBoost	Accuracy: 75% Sensitivity: 93%	
		ANN	Accuracy: 77% Sensitivity: 90%	

**NN:** Neural Network; **SVM:** Support Vector Machine; **AUC:** The area under the ROC curve serves as an indicator of the accuracy of a quantitative diagnostic test. **RF:** Random Forest; **LR:** Logistic Regression; **RF:** Random Forest; AdaBoost: Adaptive Boosting;** ANN:** Artificial Neural Network

Recent research highlights the promising role of ML in detecting and predicting ONJ by integrating clinical and radiographic data [14][24]. Table-2 outlines various ML algorithms, their input data types (clinical, radiographic, or combined), and perform¬¬ance metrics such as accuracy, sensitivity, and/or area under the curve (AUC), providing a comprehensive evaluation of each model’s effectiveness in ONJ detection or prediction. Ariji et al, [40]. developed a deep learning-based detection model using panoramic radiographs to automatically classify radiolucent lesions of the mandible. This model achieved a sensitivity of 0.88, showcasing ML's effectiveness in detecting various mandibular conditions, including dentigerous cysts. Also, Gürses et al [14]. employed an ML-based approach to detect medication-related ONJ (MRONJ) using cone beam computerized tomography (CBCT) images. They developed an SVM algorithm to differentiate between healthy and MRONJ by analyzing changes in both trabecular and cortical bone. The model achieved a high accuracy of 0.999, with strong sensitivity, specificity, and precision in identifying MRONJ. These results align with other studies that have used ML algorithms for osteonecrosis detection [39][40].

Also, Matthies et al, [15] demonstrated the U-Net models, as advanced forms of CNNs, combined with shifted-excitation Raman difference spectroscopy, can accurately differentiate antiresorptive MRONJ from viable bone, thereby enhancing both diagnosis and treatment.

Furthermore, several studies have developed ML-based models to predict osteonecrosis by analyzing complex clinical data and identifying high-risk factors and patterns [13][16][24][39][41]. For example, Reber et al, [39] compared the ML methods for the prediction of osteoradionecrosis. They reported the accuracy of the NN model (77%) is higher than other models however it is close to the SMV model (76%). On the other hand, the sensitivity of SNM is higher than the NN model. (96% Vs. 90%). Moreover, Choi et al [16]. predicted osteonecrosis via ML models by using estrogen receptor 1 polymorphism data as a novel approach.

### 6. 1. Limitations

Despite these promising developments, current research faces several limitations. A significant challenge is the availability of large, high-quality datasets. Many studies rely on small datasets, which limits the robustness and generalizability of ML models [14][22]. Additionally, while the algorithms perform well on test data, their interpretability remains a concern. Clinicians require models that not only predict outcomes but also provide clear insights into the decision-making process, an area where current ML models fall short [13][42].

Moreover, the clinical application of these models remains limited [13]. Most of the studies are still at the experiential stage, with little integration into routine clinical workflows. To bridge this gap, future research must focus on developing user-friendly tools that clinicians can readily adopt and trust in their decision-making processes.

## 7. Future Directions and Research Gaps

As ML continues to advance, the potential for improving the early detection and diagnosis of ONJ is significant [39][42]. However, several research gaps and challenges remain, particularly in the areas of data integration, dataset availability, and clinical translation.

### 7. 1. Data Integration Advances

Emerging machine learning technologies, such as federated learning and reinforcement learning, offer new opportunities for improving data integration in ONJ detection.

• Federated Learning: Traditional ML models often require centralized data, which can be challenging in healthcare due to concerns over patient privacy and data sharing [43]. Federated learning is an approach where models are trained across multiple decentralized data sources (e.g., hospitals or clinics) without sharing raw data. Instead, each institution trains a local model and only shares model updates, ensuring that patient data remains secure [44]. To utilize in the diagnosis of ONJ, federated learning could enable the integration of clinical and radiographic data from multiple institutions, leading to more robust and generalized models.

• Reinforcement Learning: While most ML applications in ONJ are based on supervised learning, reinforcement learning (RL) offers the potential for improving decision-making processes in clinical care [14][45]. In RL, the model learns by interacting with an environment and receiving feedback in the form of rewards or penalties based on its actions [46]. To detect ONJ, RL could be used to develop personalized treatment strategies, guiding clinicians on the best diagnostic and therapeutic steps based on real-time patient data.

### 7. 2. Need for Larger, Diverse Datasets

A major limitation in current ONJ research is the lack of large, well-curated datasets that combine clinical and radiographic data. ML models thrive on vast amounts of labeled data to achieve higher accuracy and generalization, yet the rarity of ONJ and the difficulties in labeling early-stage cases pose challenges [47][48].

• Curated and Labeled Datasets: The development of larger, more comprehensive datasets that combine clinical and radiographic data is essential for the future success of ML in ONJ detection.[21][40] Such datasets must include a broad range of patient demographics, clinical presentations, and imaging modalities to ensure that ML models can generalize across different populations [21].

• Diverse Data Sources: Future datasets must encompass a diversity of clinical presentations, ethnic backgrounds, and geographic regions. ONJ may present differently depending on patient comorbidities, dental health, and medication history, all of which can vary by population [13]. Collaborative efforts across global institutions are essential for generating larger, more representative datasets. Federated learning facilitates these multi-institutional collaborations by allowing data sharing across organizations without compromising patient privacy [49].

• Longitudinal Data: There is also a need for longitudinal datasets that track patients over time, allowing ML models to better predict the onset and progression of ONJ [16]. This would help address one of the key challenges in early detection, where static snapshots of clinical and radiographic data may miss subtle changes that indicate the early stages of the disease.

### 7. 3. Clinical Translation

While machine learning models have shown promising results in research, translating these technologies into clinical practice presents several challenges, particularly around ethics, explainability, and regulatory approval [21][50].

• Ethical Concerns: As ML models are increasingly used to influence clinical decisions, ethical issues such as data privacy, patient consent, and potential biases in the model become critical [51]. The use of patient data for training models raises concerns about data security where sensitive medical histories are involved. Ensuring that ML models are transparent and that patients understand how their data is being used is essential for maintaining trust in these technologies [49].

• Model Explainability: One of the primary barriers to the clinical adoption of ML models is the "black box" nature of many algorithms, particularly deep learning models [50]. While these models may offer high accuracy in detecting ONJ from radiographic images, they often provide little insight into how they arrived at a particular decision [14]. Techniques such as saliency maps, which highlight the parts of an image that contributed most to a CNN’s decision, can help make deep learning models more interpretable and thus more clinically acceptable [52].

• Regulatory Approval: Bringing ML-based diagnostic tools from research to the clinic also requires navigating complex regulatory landscapes. In most countries, medical AI systems must be rigorously validated and approved by regulatory bodies [53][54]. This process can be lengthy and requires robust evidence that the model is safe, effective, and reliable across different clinical settings. For ONJ detection, ML models must undergo extensive validation in prospective clinical trials before they can be routinely used in practice [54].

• Integration into Clinical Workflows: For ML models to be adopted in clinical settings, they must be seamlessly integrated into existing workflows. This includes ensuring that the tools are easy to use, provide real-time analysis, and complement rather than disrupt clinical decision-making [55]. For diagnosis of ONJ, ML tools must fit within the diagnostic processes used by dentists and oral surgeons, offering clear guidance without requiring extensive additional training [32][40].

## Conclusion

ML holds immense potential for enhancing the early detection and prediction of ONJ by integrating clinical and radiographic data. By analyzing complex, multimodal datasets, ML models can identify subtle patterns and early signs of ONJ that are often missed by traditional diagnostic methods. Techniques such as NNs and SVM that combine clinical risk factors with radiographic imaging have shown promise in improving diagnostic accuracy and enabling earlier intervention. However, despite these advancements, several limitations remain. The integration of heterogeneous clinical and radiographic data presents technical difficulties, including differences in data format, scale, and timing. Additionally, the scarcity of large, well-curated datasets limits the development of robust and generalizable ML models. Addressing these gaps will require concerted efforts in data standardization, the creation of longitudinal and multi-institutional datasets, and the adoption of techniques like federated learning, which can protect patient privacy while enabling collaborative research across institutions. Also, Ethical concerns, including data privacy, patient consent, and potential biases in training datasets, must be addressed to ensure that ML systems are used responsibly. Moreover, Regulatory approval presents another hurdle, as ML tools for ONJ detection require rigorous validation in clinical trials to meet safety and efficacy standards. Future research should focus on developing more interpretable ML models, expanding datasets to include more diverse populations, and integrating ML tools seamlessly into clinical workflows. There is also a need to explore emerging approaches such as federated learning, which allows the development of robust models without compromising data privacy, and reinforcement learning, which could enhance personalized treatment strategies. As researchers address the technical, ethical, and regulatory challenges, ML-driven diagnostic tools will become increasingly integrated into routine healthcare practices, enhancing the accuracy and timeliness of ONJ detection, reducing complications, and ultimately improving patient outcomes.

## Conflict of Interest

None declared.
